# A New Flow Cytometry-Based Single Platform for Universal and Differential Serodiagnosis of HTLV-1/2 Infection

**DOI:** 10.3389/fimmu.2022.795815

**Published:** 2022-04-14

**Authors:** Luciene Pimenta de Paiva, Jordana Grazziela Alves Coelho-dos-Reis, Bruno Caetano Trindade, Vanessa Peruhype-Magalhães, Márcio Sobreira Silva Araújo, Juan Jonathan Gonçalves, Ana Caroline Nogueira-Souza, Júlia Pereira Martins, Ágata Lopes Ribeiro, Ana Lucia Starling, Luiz Carlos Júnior Alcântara, Maísa Aparecida Ribeiro, Anna Bárbara de Freitas Carneiro-Proietti, Ester Cerdeira Sabino, Kelly Alves Bicalho, Andréa Teixeira-Carvalho, Olindo Assis Martins-Filho

**Affiliations:** ^1^ Instituto René Rachou, Fundação Oswaldo Cruz (FIOCRUZ)-Minas, Belo Horizonte, Brazil; ^2^ Instituto de Ciências Biológicas, Universidade Federal de Minas Gerais, Belo Horizonte, Brazil; ^3^ Faculdade de Medicina, Universidade Federal de Minas Gerais, Belo Horizonte, Brazil; ^4^ Instituto Oswaldo Cruz, Fundação Oswaldo Cruz (FIOCRUZ)/RJ, Rio de Janeiro, Brazil; ^5^ Fundação Centro de Hematologia e Hemoterapia do Estado de Minas Gerais- Hemominas (HEMOMINAS), Belo Horizonte, Brazil; ^6^ Universidade de São Paulo, Ribeirão Preto, Brazil

**Keywords:** universal and differential diagnosis, HTLV-1/2, flow cytometry, competitive assay, HTLV

## Abstract

In the present work, we developed and evaluated the performance of a new flow cytometry-based single platform, referred to as “FC-Duplex IgG1 (HTLV-1/2)”, for universal and differential serodiagnosis of HTLV-1/2 infection. The proposed technology employs a system for detection of IgG1 antibodies in a single competitive immunofluorescence platform by flow cytometry using fluorescently labeled MT-2/MoT cell line mix coupled to a highly sensitive development system (Biotin/Streptavidin/Phycoerythrin). The stability of fluorescent labeling and the antigenicity of MT-2 and MoT cell lines were confirmed upon storage at −20°C for 2, 6, and 12 months. The anti-HTLV-1/2 IgG1 reactivity, expressed as percentage of positive fluorescent cells (PPFC), was evaluated for each target antigen along the titration curve of test serum samples (1:32 to 1:4,096). Upon selection of target cell line and serum dilutions with higher segregation score between groups, the performance of “FIX” and “FIX & PERM” protocols was evaluated. The “FIX” protocol presented excellent performance indices (Se = 92%/Sp = 94%/AUC = 0.96; Se = 96%/Sp = 100%/AUC = 0.99) for the universal (HTLV-1/2 vs. NI) and differential (HTLV-1 vs. HTLV-2) diagnosis of HTLV-1 infection, respectively. Optimization of the “FIX” protocol using the principle of synchronous and asynchronous pairwise analysis further improved the performance of “FC-Duplex IgG1 (HTLV-1/2)”, using the “FIX” protocol for differential diagnosis of HTLV-1 and HTLV-2 infections (Se = 100%/Sp = 100%/AUC = 1.00). In conclusion, the “FC-Duplex IgG1 (HTLV-1/2)” method represents an innovation in the biotechnology segment with the potential to compose a serological kit for differential diagnosis of HTLV-1/2 infection for reference laboratories and blood centers.

## Introduction

The human T-cell lymphotropic virus (HTLV) is a retrovirus with a global distribution. The HTLV is classified into four distinct types, HTLV-1, HTLV-2, HTLV-3, and HTLV-4 (1). While HTLV-1/2 infections represent a high-risk factor for lymphoproliferative/inflammatory diseases, HTLV-3 and HTLV-4 have not been linked to clinical illnesses (1).

The HTLV-1/2 infections are estimated to affect approximately 10–20 million people worldwide, with prevalence rates ranging from 5% to 27% (2). HTLV-1 infection is endemic in Japan, South America, Caribbean Islands, and Sub-Saharan Africa, whereas HTLV-2 infection is reported in pygmy communities in Central Africa and indigenous populations from the Americas (3, 4).

The HTLV-1 infection has been associated with adult T-cell leukemia/lymphoma and HTLV-1-associated myelopathy/tropical spastic paraparesis (HAM/TSP) (5, 6), in addition to other secondary comorbidities and/or opportunistic infections (7). On the other hand, although the HTLV-2 infection has been identified in patients with hairy cell leukemia (8, 9), no clinical correlation between HTLV-2 and lymphoproliferative disease has been established (10). Moreover, the epidemiology of HTLV-2 infection is also distinct, being primarily detected on indigenous populations and drug users (3, 4, 11). Based on these clinical and epidemiological aspects, the differential diagnosis of HTLV-1 and HTLV-2 infections is relevant.

The diagnostic methods for HTLV1/2 infection include an initial screening test, usually enzyme-linked immunosorbent assay (ELISA), chemiluminescence enzyme-linked immunoassay (CLEIA), or particle agglutination (PA), followed by a confirmatory test, including Western blot (WB), innogenetics line immunoassay (INNO-LIA), as well as qualitative and/or quantitative polymerase chain reaction (PCR). In general, the serological diagnosis of HTLV infection is based on the detection of specific antibodies to different HTLV antigens (12, 13). However, due to the relatively high homology between HTLV-1 and HTLV-2, the current serological methods applied for screening assay detect antibodies to both viruses with consequent serological cross-reactivity (14).

The differential diagnosis between HTLV-1 and HTLV-2 infections can be achieved by molecular methods. However, molecular approaches are expensive, time-consuming, and labor-intensive, requiring specialized laboratory infrastructure. In this sense, antibody tests are the best option for routine diagnostic purposes (12). The availability of simple serological assays capable to differentiate HTLV-1 from HTLV-2 infection would therefore be most useful for counseling HTLV-seropositive donors detected in blood banks and also while performing seroepidemiological studies (15). The WB has the perspective of typing for HTLV-1 and HTLV-2. Despite improvements in WB assay specificity, by using specific recombinant HTLV-1/2 Env glycoproteins, indeterminate serological patterns still represent a relevant concern in WB results (16, 17).

In this scenario, there is still no gold standard method for differential diagnosis of HTLV-1 and HTLV-2 infections to meet the needs of blood centers and clinical laboratories (18). In general, differential diagnosis of HTLV-1 and HTLV-2 infections requires the use of multiple assays using distinct serological and molecular platforms. Therefore, the search for a single step approach for differential laboratorial diagnosis of HTLV-1/2 is a challenge for the scientific community.

Previous studies from our group have demonstrated the applicability and accuracy of flow cytometry-based methods for detecting specific anti-HTLV-1 IgG1 antibodies (19, 20). Aiming at optimizing a single-step serological procedure to differentiate the infection with HTLV-1 and HTLV-2 types, in the present study, we have developed an innovative flow cytometry assay, referred to as “FC-Duplex IgG1 (HTLV-1/2)”, for universal and differential diagnosis of HTLV infections. The proposed technology employs a system for detection of IgG1 antibodies in a single competitive immunofluorescence platform by flow cytometry, using fluorescent-labeled stained antigenic support (MT-2/MoT cell line Mix) coupled to a high-sensitivity development system (Biotin/Streptavidin/Phycoerythrin). The use of synchronous and asynchronous pairwise analysis has improved the performance of “FC-Duplex IgG1 (HTLV-1/2)” for differential diagnosis of HTLV infection.

## Materials and Methods

### Study Population and Ethical Approval

The Interdisciplinary Research Group on HTLV—GIPH—is an open cohort, started in March 1997, which is a world reference to evaluate the natural history of HTLV infection.

The present investigation included 135 subjects from the GIPH cohort, comprising patients with positive serology for HTLV-1/2 (*n* = 114) and non-infected controls (NI, *n* = 21). The HTLV-1/2 group was composed of patients of both genders, aged from 18 to 75 years old, under medical care by one of us (AS) at the Hospital das Clínicas of the Universidade Federal de Minas Gerais—UFMG. The HTLV-1/2 patients with positive serology for hepatitis B or C, Chagas disease, syphilis, and HIV infection, and drug use history in the preceding 6 months or in the use of immunomodulatory therapy were excluded. Based on molecular diagnosis, the HTLV-1/2 group was further classified as HTLV-1 (*n* = 88) and HTLV-2 (*n* = 26) patients with confirmatory diagnosis by RT-qPCR. During clinical and neurological examinations, the HTLV-1 group was further categorized into three subgroups, considering the HAM diagnosis criteria according to De Castro-Costa et al. (21), and using the Expanded Disability Status Scale (EDSS) (22) and OSAME scale (23). Based on these criteria, HTLV-1 patients were classified as follows: HTLV-1 Asymptomatic Carriers with EDSS and OSAME scores = 0 (HAC, *n* = 27); HTLV-1 with putative HAM status that did not fulfill the De Castro-Costa criteria, i.e., EDSS and OSAME scores = 1 to 2 (pHAM, *n* = 32); and patients with definite diagnosis of HTLV-1-associated myelopathy/tropical spastic paraparesis, with EDSS and OSAME scores higher than 2 (HAM, *n* = 29).

The NI control group comprised blood donors, both genders, aged from 18 to 50 years old, with negative serology for infectious diseases tested during screening at Fundação HEMOMINAS.

This study was approved by the Research Ethics Committee of the René Rachou Institute - FIOCRUZ/MG (C.A.A.E. N° 15047313.8.0000.5091). All participants have signed a written informed consent and the study followed the guidelines and standards for research involving human beings, according to the resolution 466/2012 from the Brazilian National Health Council.

Whole blood samples (10 ml) were collected without anticoagulant from each participant to obtain serum samples. Serum was aliquoted and stored in −80°C until processing by FC-Duplex IgG1 (HTLV-1/2) assay.

### Flow Cytometric “FC-Duplex IgG1 (HTLV-1/2)” Assay

#### Antigen Support Preparation—MT-2 and MoTCell Lines

Stocks of MT-2 and MoT cell lines, permanently infected by HTLV-1 and HTLV-2, respectively, were used as antigen support for Flow Cytometric “FC-Duplex IgG1 (HTLV-1/2)” assay. The MT-2 cell line was obtained from the Bio-Manguinhos, FIOCRUZ/RJ. This cell line was originally obtained from co-culture of cells from a patient with adult T-cell leukemia/lymphoma (ATLL) and umbilical cord lymphocytes (24, 25). The MT-2 cell line contains HTLV-1 integrated into the cellular genome.

The MoT cell line was kindly provided by Dr. Steven Jacobson from the National Institutes of Health (NIH, USA). This cell line was originally obtained from a patient infected with the HTLV-2 with hairy cell leukemia (8).

Both cell lines were maintained and cryopreserved in liquid nitrogen at Laboratório de Virologia Básica e Aplicada, Instituto de Ciências Biológicas, Universidade Federal de Minas Gerais. All procedures were performed in Level 2 biosecurity laboratory. Bulk-culture batches were carried out for large-scale production of MT-2 and MoT antigen supports for Flow Cytometric “FC-Duplex IgG1 (HTLV-1/2)” assay. For this purpose, MT-2 and MoT aliquots cells were thawed and seeded (approximately 1.0 × 10^6^ cells/ml) into RPMI medium supplemented with 20% of fetal bovine serum (FBS) using 75-cm^2^ tissue culture bottles. The cultures were maintained at 37°C, 5% CO_2_, 95% in a humidified incubator for 72 h. At each passage, cell viability was estimated by Trypan Blue staining, using the automated cell counter (Countess^®^, Thermo Fisher Scientific, Waltham, MA, USA). The cultures in log phase were maintained by continuous passages to obtain bulk batches (1.0 × 10^9^ cells). The cells were homogenized and submitted to differential centrifugation at 80 × *g* for 10 min at room temperature to eliminate cell clumps and recover the single-cell suspension in the supernatant. The single-cell suspensions were washed twice with phosphate buffered saline (PBS) supplemented with 0.5% FBS (PBS 0.5% FBS) by centrifugation at 400 × *g* for 10 min at 4°C. The cell suspension was adjusted to 1.0 × 10^6^ cells/ml in PBS.

The MT-2 and MoT single-cell suspensions were submitted to the “FIX” protocol, as previously described by Coelho-Dos-Reis et al. (20), modified as follows: Aliquots of single-cell suspensions (1.0 × 10^6^ cells/ml) were mixed with equal volume of FACS Fix Solution (10 g/L of paraformaldehyde; 10.2 g/L of sodium cacodylate; 6.63 g/L of sodium chloride, pH 7.2) and incubated overnight at 4°C. The “FIX” cell suspensions were washed once with PBS (400 × *g*, 10 min, 4°C) and maintained at 4°C until use in the “FIX” protocol.

In parallel, aliquots of MT-2 and MoT single-cell suspensions were submitted to the “FIX & PERM” protocol, as previously described by Coelho-Dos-Reis et al. (20), modified as follows: aliquots of “FIX” cells (1.0 × 10^6^ cells/ml in PBS) were centrifuged and resuspended in equal volume of PBS supplemented with 0.5% bovine serum albumin plus 0.1% sodium azide, 0.5% saponin (PBS-P) and incubated for 10 min at room temperature. Following the “FIX & PERM”, cells were washed once (400 × *g*, 10 min, 4°C) with PBS supplemented with 0.5% bovine serum albumin plus 0.1% sodium azide (PBS-W) and maintained at 4°C until use in the “FIX & PERM” protocol.

The morphometric profiles of MT-2 and MoT cell lines, submitted to both protocols (“FIX” and “FIX & PERM”) were monitored by flow cytometry, using the size [Forward Scatter (FSC)] and granularity [Side Scatter (SSC)] parameters.

#### Differential Fluorescent Staining of Antigen Support—MT-2 and MoT Cell Lines

The MT-2 and MoT cell lines used in both protocols, “FIX” and “FIX & PERM”, were previously submitted to a protocol of differential fluorescent staining using distinct concentrations of one fluorescent dye: either fluorescein isothiocyanate-FITC (Sigma Aldrich, MO, USA) or Alexa Fluor 647 Triethylammonium Salt (Thermo Fisher Scientific, MA, USA). For this purpose, aliquots of “FIX” and “FIX & PERM” cells (1.0 × 10^6^ cells/ml in PBS) were incubated for 30 min, at 37°C with FITC (at a final concentration of 0.001 µg/ml for MT-2 and 0.01 µg/ml for MoT) or Alexa Fluor 647 (at a final concentration of 0.002 µg/ml for MT-2 and 0.04 µg/ml for MoT) in serum-free PBS. The dye concentrations were determined by pre-testing several concentrations (data not shown). No pre-activation of dyes with an amine or thiol group was required. After incubation, the stained cell suspensions were washed once with serum free PBS (400 × *g*, 10 min, 4°C) and submitted to quality control assessment by flow cytometry.

**Figure 1:**
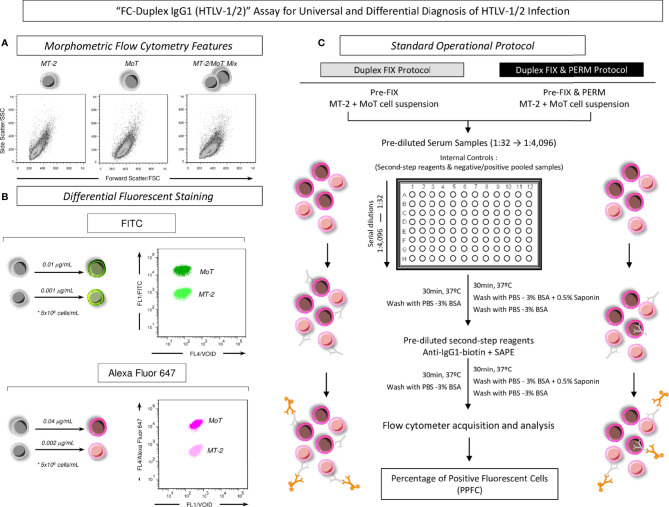
Methodological outline of “FC-Duplex IgG1 (HTLV-1/2)” assay for universal and differential diagnosis of HTLV-1/2 infection. **(A)** Morphometric flow cytometry features of MT-2, MoT, and MT-2/MoT Mix underscoring the overlapping distribution of cell lines on Forward Scatter—FSC (size) vs. Side Scatter—SSC (granularity) flow cytometric plots. **(B)** Differential fluorescent staining of MT-2 and MoT cell lines with FITC and Alexa Fluor 647 allowed the segregation of MT-2 and MoT cell lines according to their fluorescence intensity, with MoT cell line exhibiting higher fluorescence intensity. **(C)** Standard operating procedure for “FIX” and “FIX & PERM” protocols using Alexa Fluor 647-labeled MT-2/MoT Mix underscoring the four major steps, including (i) incubation of the MT-2 and MoT Mix with serial dilution of heat-inactivated test serum samples (1:32 to 1:4,096); (ii) washing steps and incubation with biotin-labeled human anti-IgG1 plus Phycoerythrin-labeled Streptavidin-SAPE; (iii) washing steps and final fixation prior to flow cytometric acquisition; and (iv) data report as percentage of positive fluorescent cells (PPFC).

The fluorometric profile of stained MT-2 and MoT cell lines, submitted to both protocols (“FIX” and “FIX & PERM”) were monitored by flow cytometry as quality control, using the FL1 (FITC) and FL4 (Alexa Fluor 647) parameters. Immediately prior to use in the “FC-Duplex IgG1 (HTLV-1/2)” assay, a 1:1 mixture of FITC or Alexa Fluor 647-labeled MT-2 and MoT cell lines (Mix MT-2/MoT) was prepared with 50% proportion and monitored by flow cytometry using the FL1 (FITC) and FL4 (Alexa Fluor 647) parameters. Autofluorescence of unstained MT-2 and MoT cell lines was monitored prior to the fluorescent staining procedure. **Figure 1** illustrates the morphometric flow cytometry features of MT-2, MoT, and MT-2/MoT mix (**Figure 1A**) and the differential fluorescent staining of MT-2 and MoT cell lines (**Figure 1B**).

#### Standard Operating Procedure for the “FC-Duplex IgG1 (HTLV-1/2)” Assay

In a pre-analytical step, serum aliquots maintained at −80°C were thawed at 37°C, inactivated at 56°C for 30 min, and centrifuged at 14,000 rpm at 4°C for 5 min to remove debris. Fifty 50-μl aliquots of heat inactivated serum were transferred to “U” bottom 96-well plates (Nunc, Roskilde, Denmark) and submitted to twofold serial dilution (1:32 to 1:4,096) in PBS-3% FBS. Subsequently, each well received 50 μl of FITC or Alexa Fluor 647-labeled MT-2/MoT mix (5.0 × 10^4^ cells/well), accordingly for the “FIX” and “FIX & PERM” protocols. The plate was homogenized with circular movements and incubated for 30 min at 37°C. After incubation, the MT-2/MoT mix was washed with 100 μl and subsequently with 150 μl of PBS-3% PBS (400 × *g*, 10 min, 4°C), the supernatant was discarded, and the pellet was homogenized. Thereafter, 50 μl of biotin-labeled human anti-IgG1, clone 8c/6-39 (1:6,400), plus 10 μl of Phycoerythrin-labeled Streptavidin-SAPE (1: 400) were added as secondary reagents to each well, previously diluted with PBS-3% FBS or PBS-P for the “FIX” and “FIX & PERM” protocols, respectively. The plates were incubated for 30 min at 37°C, and the MT-2/MoT mix was washed twice, as described above. After centrifugation, the supernatant was discarded, the pellet was homogenized, and 200 μl of FACS Fix Solution was added to each well. The plates were kept at 4°C, protected from light, up to 24 h prior to acquisition in the flow cytometer. Internal controls of nonspecific binding of secondary reagents were carried out for each experimental batch. **Figure 1C** illustrates the standard operating procedure for “FIX” and “FIX & PERM” protocols using the Alexa Fluor 647-labeled MT-2/MoT mix.

#### Flow Cytometric Acquisition

Following the standard operating procedure for the “FC-Duplex IgG1 (HTLV-1/2)” assay, the MT-2 and MoT Mix incubated with the test serum samples was acquired in a FACSCalibur Flow Cytometer (BD, Bioscience, San Jose, CA, USA) equipped with Argon (488 nm) and HeNe (633 nm) lasers using previously defined instrument settings for FSC (size) and SSC (granularity), both in linear scales, as follows: FSC = E00; SSC = 300. The threshold was set on FSC = 200 to minimize noise interference during acquisition. The fluorescence parameters (FL1, FL2, and FL4) were set on *log* scale with compensation systems activated, based on the instrument settings obtained during calibration using CaliBrite^®^ kit (BD Biosciences San Diego, CA, USA). A total of 10,000 events were acquired per sample and data were stored using the CellQuest™ software package (BD, Bioscience, San Jose, CA, USA).

#### Analysis of Anti-HTLV-1/2 IgG1 Reactivity

**Figure 2:**
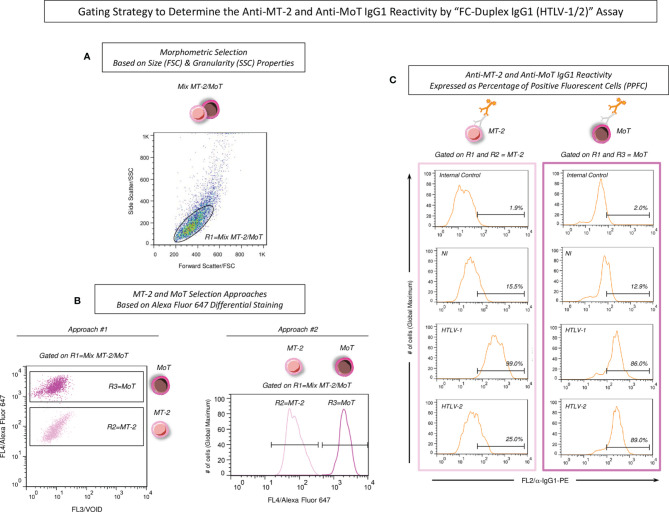
Gating strategy to determine the anti-MT-2 and anti-MoT IgG1 reactivity by “FC-Duplex IgG1 (HTLV-1/2)” assay. **(A)** MT-2 and MoT Mix first gated based on the homogeneous morphometric Forward Scatter—FSC (Size) vs. Side Scatter—SSC (Granularity) distribution (R1). **(B)** MT-2 and MoT cell lines selection based on Alexa Fluor 647 differential staining, carried out using two alternative approaches, including Approach # 1—bi-dimensional FL3 (Empty Channel-VOID) versus FL4 (Alexa Fluor 647) dot plot distribution or Approach # 2—uni-dimensional FL4 (Alexa Fluor 647) histogram. **(C)** The anti-MT-2 and anti-MoT IgG1 reactivity reported as percentage of positive fluorescent cells (PPFC), considering the shift of FL2 intensity (α-IgG1-biotin/SAPE) outside the positivity limit (PPFC < 2%) established for the internal control of secondary reagents.

**Figure 2** displays the gating strategies and the following procedures to determine the anti-HTLV-1/2 IgG1 reactivity by the “FC-Duplex IgG1 (HTLV-1/2)” Assay as illustrated for the Alexa Fluor 647-labeled MT-2/MoT Mix. The analysis of the anti-MT-2/MoT IgG1 reactivity was performed by first gating the MT-2 and MoT Mix based on their FSC versus SSC properties (**Figure 2A**, R1), followed by further selection of MT-2 and MoT cell lines (**Figure 2B**, R2 and R3), based on their differential fluorescent labeling with Alexa Fluor 647 using the FL4 parameter. For this purpose, two strategies were alternatively used, namely, Approach # 1—bi-dimensional FL3 (Empty Channel-VOID) versus FL4 (Alexa Fluor 647) dot plot distributions, or Approach # 2—unidimensional FL4 (Alexa Fluor 647) histograms. Upon selective cell lines gating, unidimensional FL2 (α-IgG1-biotin/SAPE) histograms were constructed to assess the anti-HTLV-1/2 IgG1 reactivity. The anti-HTLV-1/2 IgG1 reactivity was expressed as percentage of positive fluorescent cells (PPFC) determined based on the shift of FL2 (α-IgG1-biotin/SAPE) outside the positivity limit (PPFC < 2%) established for the internal control of secondary reagents (**Figure 2C**). The FlowJo 10.0.1 software (TreeStar, San Diego, CA, USA) was used for gating strategies and analysis of anti-MT-2 and anti-MoT IgG1 reactivities.

#### Stability of Fluorescent Staining and Antigenicity of MT-2 and MoT Cell Lines

The stability of differential fluorescent labeling and antigenicity of MT-2 and MoT cell lines were monitored under storage at −20°C for 2, 6, and 12 months, as a quality control criterion of reagent storage for the “FC-Duplex IgG1 (HTLV-1/2)” Assay. For this purpose, cells were fixed (“FIX” protocol) or fixed and permeabilized (“FIX & PERM” protocol), stained, and then stored. In detail, aliquots of Alexa Fluor 647-labeled MT-2 and MoT, prepared using the “FIX” or “FIX & PERM” protocols were mixed in a 1:1 ratio and immediately frozen in cryovials at −20°C, without addition of any cryopreservation solutions, at the maximum concentration of 10 × 10^6^ mixed cells per vial. The fluorometric profile of differential labeling was monitored at 2, 6, and 12 months after freezing. For that, cells were thawed at water bath at 37°C and resuspended in PBS 1× for flow cytometric acquisition. In parallel, the antigenicity stability of MT-2 and MoT was also monitored using standard operating procedure for the “FC-Duplex IgG1 (HTLV-1/2)” assay.

### Performance Analysis of the “FC-Duplex IgG1 (HTLV-1/2)” Assay

The performance of the “FC-Duplex IgG1 (HTLV-1/2)” assay using the “FIX” and “FIX & PERM” protocols was assessed along the titration curves (1:32 to 1:4,096) using individual samples from NI vs. HTLV-1/2 for universal diagnosis as well as HTLV-1 vs. HTLV-2 for differential diagnosis. Distinct approaches were employed to define specific serum dilutions and cutoff values as well as to determine the performance indices (sensitivity, specificity, and global accuracy), including scatter plot distribution of individual PPFC values, Two-Graph Receiver Operating Characteristic plot (TG-ROC), and ROC curve analysis. Optimized performance of the “FC-Duplex IgG1 (HTLV-1/2)” assay was further assessed using synchronous and asynchronous strategies of pairwise serum dilutions. The MedCalc version 7.3.0.0 (MedCalc Software Ltd, Ostend, Flandres Ocidental, BE) was employed for performance analysis. Prisma GraphPad software version 5.0 (GraphPad Prism Software, San Diego, CA, EUA) was used for graphical arts.

## Results

### Differential Fluorescent Staining of MT-2 and MoT Cell Lines

**Figure 1A** illustrates the morphometric and fluorometric profiles of fluorescent-stained MT-2 and MoT cell lines, employed as target antigens in the flow cytometric assay for selective analysis of anti-HTLV1/2 IgG1. The morphometric flow cytometry features demonstrated that MT-2 and MoT cell lines exhibited similar profiles, with overlapping properties regarding the FSC and SSC parameters. However, the differential fluorescent staining approaches using distinct concentrations of FITC or Alexa Fluor 647 were able to segregate the MT-2 and MoT cell lines according to their fluorescence intensity (**Figure 1B**). In both FITC or Alexa Fluor 647 staining protocols, the MoT cell line exhibited higher fluorescence intensity, while the MT-2 cell line displayed lower fluorescence intensity. This result clearly demonstrates that MT-2 and MoT cell lines clustered apart at distinct regions on the bidimensional fluorescence dot plot distributions (**Figure 1B**). Noteworthy was that Alexa Fluor 647 staining yielded higher segregation profile between MT-2 and MoT cell lines (**Figure 1B**). Therefore, the Alexa Fluor 647 differential staining was selected for further use in the standard operating protocol (**Figure 1C**).

### Stability of Fluorescent Staining and Antigenicity of MT-2 and MoT Cell Lines

**Figure 3:**
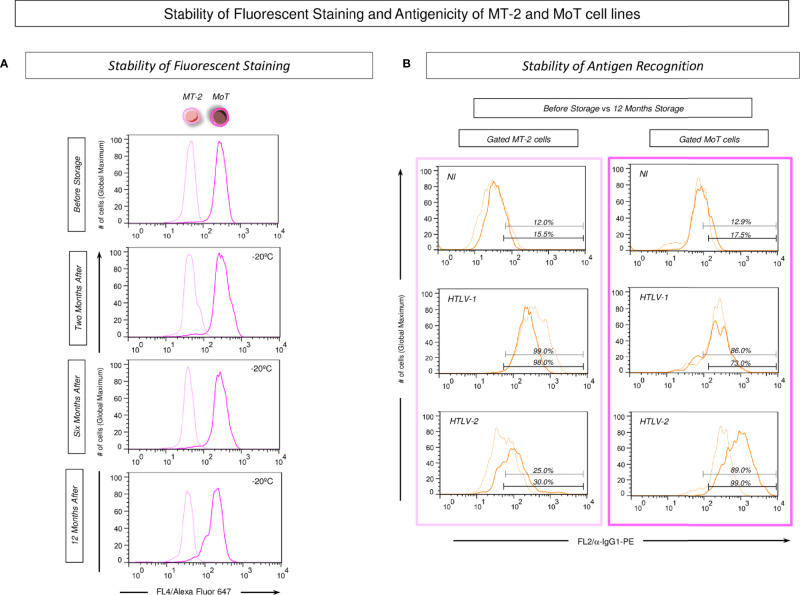
Stability of fluorescent staining and antigenicity of MT-2 and MoT cell lines. **(A)** The staining stability of Alexa Fluor 647 fluorescent-labeled MT-2 and MoT cell lines was monitored upon storage at −20°C for 2, 6, and 12 months. Data reported as overlaid histogram of fluorescent intensity for Alexa Fluor 647 labeling illustrating that along the storage timeline, the MoT cell line exhibited higher fluorescence intensity (dark pink) than the MT-2 cell line (light pink). **(B)** Stability of antigen recognition on MT-2 and MoT cell lines stored up to 12 months at −20°C. Data reported as overlaid histograms for antigen recognition by poolled serum samples from NI, HTLV-1, and HTLV-2 patients illustrating the stability of antigen recognition prior storage (light orange) vs. 12 months storage at −20°C (dark orange).

The stability of fluorescent stained MT-2 and MoT cell lines was evaluated upon selective gating strategies as described in methods (**Figures 2A, B**). For this purpose, the Alexa Fluor 647 fluorescent-stained MT-2 and MoT cell lines were monitored upon storage at −20°C for 2, 6, and 12 months (**Figure 3A**). The results demonstrated that the fluorescent staining remained stable up to 12 months as compared to the fluorescent profile observed prior to storage (**Figure 3A**).

Additional analysis was carried out to monitor the stability of antigen recognition on MT-2 and MoT cell lines stored up to 12 months at −20°C (**Figure 3B**). The results showed that MT-2 and MoT cell lines were similarly recognized by pools of serum samples from non-infected controls (NI) as well as HTLV-1 and HTLV-2 patients even up to 12 months storage at −20°C as compared to the profile observed prior storage (**Figure 3A**).

### Performance of the “FC-Duplex IgG1 (HTLV-1/2)” Assay for Universal Diagnosis

**Figure 4:**
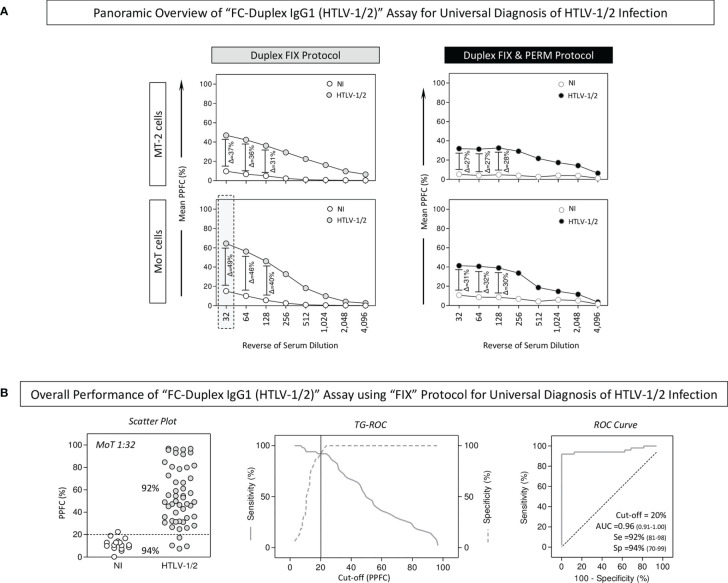
Applicability of “FC-Duplex IgG1 (HTLV-1/2)” assay for universal diagnosis of HTLV-1/2 infection. **(A)** Panoramic overview of “FIX” and “FIX & PERM” protocols according to the overall anti-MT-2 and anti-MoT IgG1 mean reactivities along the titration curves (1:32 to 1:4,096) used to identify the target cell line and the serum dilution with higher segregation score (Δ = delta reactivity) between NI vs. HTLV-1/2 groups (dashed rectangle). **(B)** Overall performance of “FC-Duplex IgG1 (HTLV-1/2)” assay for universal diagnosis of HTLV-1/2 infection assessed by scatter plot distribution of individual values of IgG1 reactivity, TG-ROC parameters, and ROC curve analysis using the selected cell line and the pair of attributes “serum dilution/cutoff” (MoT 1:32/PPFC = 20%) from the “FIX” protocol.

The performance of the “FC-Duplex IgG1 (HTLV-1/2)” assay was assessed for universal diagnosis of HTLV-1/2 infection using both “FIX” and “FIX & PERM” protocols (**Figure 4**). For this purpose, the overall anti-MT-2 and anti-MoT IgG1 mean reactivities were evaluated along the titration curves (1:32 to 1:4,096) to first identify the serum dilution with higher segregation score (Δ = delta reactivity) between NI vs. HTLV-1/2 groups (**Figure 4A**). The results demonstrated that anti-MoT cells’ IgG1 reactivity at 1:32 serum dilution, using the “FIX” protocol, led to higher delta reactivity between NI and HTLV-1/2 groups. These parameters were then selected for further performance assessment (**Figure 4A**, dashed rectangle).

Scatter plot distribution of individual values of IgG1 reactivity, TG-ROC parameters, and ROC curve analysis indicated that a single pair of attributes “serum dilution/cutoff” (MoT 1:32/PPFC = 20%) using the “FIX” protocol presented excellent performance indices (Se = 92%; Sp = 94%; and AUC = 0.96) for the universal diagnosis of HTLV-1 infection (**Figure 4B**).

### Performance of “FC-Duplex IgG1 (HTLV-1/2)” Assay for Differential Diagnosis

**Figure 5:**
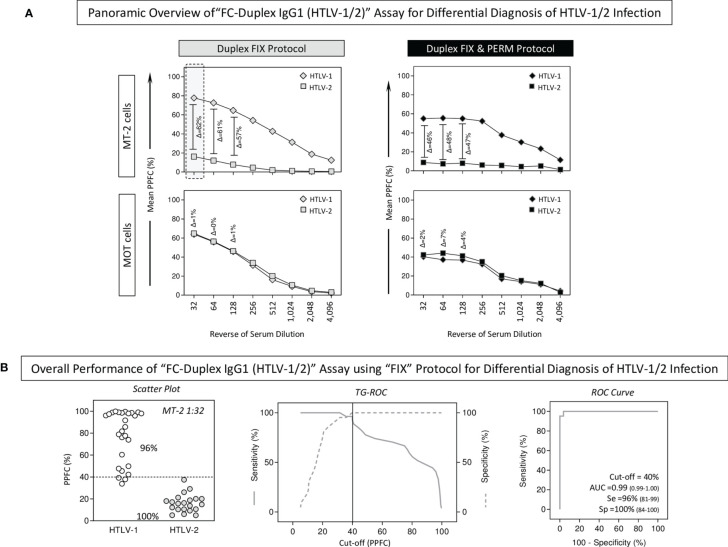
Applicability of “FC-Duplex IgG1 (HTLV-1/2)” assay for differential diagnosis of HTLV-1/2 infection. **(A)** Panoramic overview of “FIX” and “FIX & PERM” protocols according to the overall anti-MT-2 and anti-MoT IgG1 mean reactivities along the titration curves (1:32 to 1:4,096) used to identify the target cell line and the serum dilution with higher segregation score (Δ = delta reactivity) between HTLV-1 vs. HTLV-2 groups (dashed rectangle). **(B)** Overall performance of “FC-Duplex IgG1 (HTLV-1/2)” assay for differential diagnosis of HTLV-1/2 infection assessed by scatter plot distribution of individual values of IgG1 reactivity, TG-ROC parameters, and ROC curve analysis using the selected cell line and pair of attributes “serum dilution/cutoff” (MT-2 1:32/PPFC = 40%) from the “FIX” protocol.

The performance of the “FC-Duplex IgG1 (HTLV-1/2)” assay was further assessed for differential diagnosis of HTLV-1 from HTLV-2 infections using both “FIX” and “FIX & PERM” protocols (**Figure 5**). For this purpose, the overall anti-MT-2 and anti-MoT IgG1 mean reactivities were evaluated along the titration curves (1:32 to 1:4,096) to first identify the serum dilution with higher segregation score (Δ = delta reactivity) between HTLV-1 vs. HTLV-2 subgroups (**Figure 5A**). The results demonstrated that anti-MT-2 cells’ IgG1 reactivity at 1:32 serum dilution, using the “FIX” protocol, led to higher delta reactivity between HTLV-1 and HTLV-2 subgroups. Therefore, these parameters were then selected for further performance assessment (**Figure 5A**, dashed rectangle).

The analysis of scatter plots of individual IgG1 reactivity together with TG-ROC and ROC curve profiles indicated that a single pair of attributes “serum dilution/cutoff” (MT-2 1:32/PPFC = 40%) using the “FIX” protocol presented outstanding performance indices (Se = 96%; Sp=100%; and AUC = 0.99) for the differential diagnosis of HTLV-1 and HTLV-2 infections (**Figure 5B**).

### Optimization of the “FC-Duplex IgG1 (HTLV-1/2)” Assay Using the “FIX” Protocol for Differential Diagnosis

**Figure 6:**
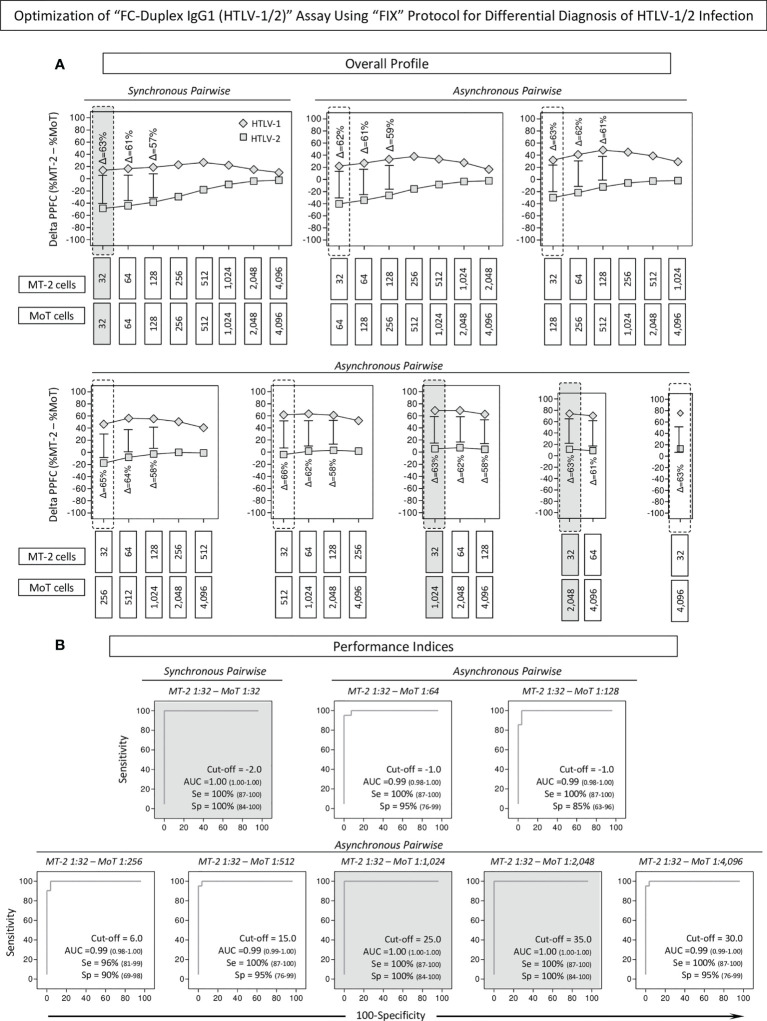
Optimization of “FC-Duplex IgG1 (HTLV-1/2)” assay using “FIX” Protocol for differential diagnosis of HTLV-1/2 infection. **(A)** Overall profile of IgG1 reactivity calculated as differential reactivity between anti-MT-2 and anti-MoT cell lines (Delta PPFC = %MT-2 – %MoT) along the titration curves (1:32 to 1:4,096) using synchronous and asynchronous pairwise serum dilutions, underscoring the best pairwise conditions based on the higher delta reactivity to segregate HTLV-1 from HTLV-2 subgroups (dashed gray background). **(B)** Performance indices of synchronous and asynchronous pairwise serum dilutions to segregate HTLV-1 from HTLV-2 subgroups assessed by ROC curve analysis. Pairwise conditions with outstanding performance indices (Se = 100%; Sp = 100%; and AUC = 1.00) for the differential diagnosis of HTLV-1 and HTLV-2 infections are underscored by gray background.

Aiming at further optimizing the performance of the “FC-Duplex IgG1 (HTLV-1/2)” assay using the “FIX” protocol for differential diagnosis of HTLV-1 from HTLV-2 infections, the overall profile of IgG1 reactivity was calculated as the differential reactivity between anti-MT-2 and anti-MoT cell lines (Delta PPFC = %MT-2 – %MoT) along the titration curves (1:32 to 1:4,096), using synchronous and asynchronous pairwise serum dilutions (**Figure 6**). The simultaneous analysis of the magnitude of the differential reactivity between anti-MT-2 and anti-MoT cell lines (ΔPPFC, **Figure 6A**) and the Area Under the ROC Curve (AUC, **Figure 6B**) data analysis demonstrated that [MT-2 1:32 – MoT 1:32], [MT-2 1:32 – MoT 1:64], and [MT-2 1:32 – MoT 1:128] were the best pairwise conditions with higher ΔPPFC and AUC to segregate HTLV-1 from HTLV-2 subgroups (**Figures 6A, B**, dashed gray background). The results indicated that synchronous pairwise [MT-2 1:32 – MoT 1:32] with Delta PPFC cutoff = 2.0 presented outstanding performance indices (Se = 100%; Sp = 100%) for the differential diagnosis of HTLV-1 and HTLV-2 infections (**Figure 6B**). Similarly, the asynchronous pairwise [MT-2 1:32 – MoT 1:1,024] with Delta PPFC cutoff = 25.0 as well as [MT-2 1:32 – MoT 1:2,048] with Delta PPFC cutoff = 35.0 also presented outstanding performance indices (Se = 100%; Sp = 100%) for the differential diagnosis of HTLV-1 and HTLV-2 infections (**Figure 6B**).

### Performance of the Optimized “FC-Duplex IgG1 (HTLV-1/2)” Assay for Differential Diagnosis

**Figure 7:**
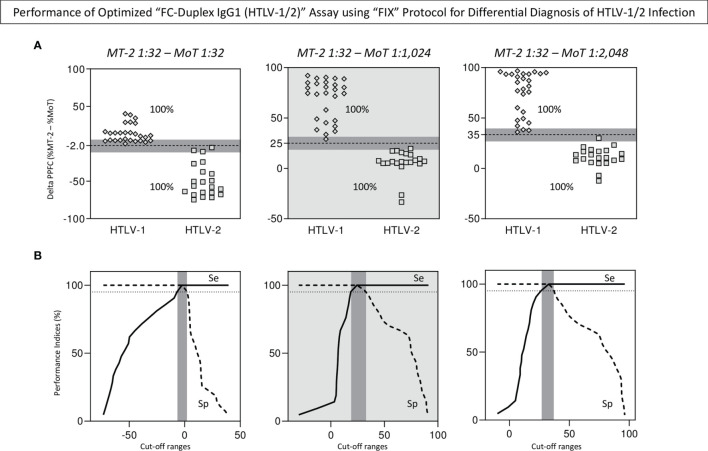
Performance of optimized “FC-Duplex IgG1 (HTLV-1/2)” assay for differential diagnosis of HTLV-1/2 infection. **(A)** Scatter distribution of individual values obtained of synchronous [MT-2 1:32 – MoT 1:32] and asynchronous pairwise calculations [MT-2 1:32 – MoT 1:1,024] and [MT-2 1:32 – MoT 1:2,048] used to define the best condition for differential diagnosis of HTLV-1 and HTLV-2 infections, according to the number of samples confined into the 10% gray zone of selected cutoff values (gray background). **(B)** TG-ROC curves used to define the higher valid cutoff ranges (95% CI for Se and Sp) selected as the most reliable pairwise condition to segregate HTLV-1 from HTLV-2 subgroups (gray background).

Aiming at defining the best pairwise condition to segregate HTLV-1 from HTLV-2 subgroups, the performance of selected synchronous [MT-2 1:32 – MoT 1:32] and asynchronous pairwise [MT-2 1:32 – MoT 1:1,024] and [MT-2 1:32 – MoT 1:2,048] was further evaluated based on the scatter distribution of individual values, considering the number of samples confined into the 10% gray zone of selected cutoff values (**Figure 7A**). Additionally, data were analyzed according to the valid cutoff ranges (95% CI for Se and Sp) calculated by the TG-ROC parameters (**Figure 7B**). The results demonstrated that the asynchronous pairwise [MT-2 1:32 – MoT 1:1,024] fulfill both criterion and was selected as the most reliable pairwise condition to segregate HTLV-1 from HTLV-2 subgroups with outstanding performance (**Figure 7**).

### “FC-Duplex IgG1 (HTLV-1/2)” Assay for Prognosis of HTLV-1 Infection

Considering the relevance of antibody quantification as a prognosis biomarker for HTLV-1 clinical progression from asymptomatic cases (HAC) towards HAM status, the performance of the “FC-Duplex IgG1 (HTLV-1/2)” assay and that of both “FIX” and “FIX & PERM” protocols were evaluated, and the data are presented in **Supplementary Figure 1**. The results demonstrated that anti-MoT cells’ IgG1 reactivity at 1:256 serum dilution, using the “FIX” protocol, led to higher delta reactivity between HAC and HAM groups (**Supplementary Figure 1A**). Further analysis of the selected parameters “serum dilution/cutoff” (MT-2 1:256/PPFC = 60%) revealed that the “FC-Duplex IgG1 (HTLV-1/2)” assay displayed moderate overall performance (Se = 89%; Sp = 89%; and AUC = 0.99) when applied with prognosis purposes of HTLV-1 infection to segregate HAC from HAM (**Supplementary Figure 1B**). Additional analysis of samples from patients with putative HAM status (pHAM) demonstrated that 60% of them displayed a reactivity profile similar to that observed in HAC and 40% already presented higher anti-MT-2 reactivity, characteristic of HAM (**Supplementary Figure 1B**).

## Discussion

The HTLV-1/2 is a public health problem, representing a silent global challenge with estimates of at least 20 million people infected with HTLV-1 around the globe (2). The HTLV-1/2 infection is endemic in several countries and also reported in indigenous communities in Africa and the Americas (3, 4). Besides the differences in the epidemiological features of HTLV-1 and HTLV-2 infections, distinct clinical manifestations have been associated with these viral infections, with HTLV-1 infection being associated with onco-hematological diseases and inflammatory conditions, other secondary comorbidities, and/or opportunistic infections (5, 6), whereas HTLV-2 infection rarely correlated with lymphoproliferative diseases (8–10).

Noteworthy is the importance of these viruses not only due to the distinct epidemiological and the clinical features, but also by the fact that the differential diagnosis of these viral infections still represents a challenge for clinical laboratories. Currently, the diagnosis of HTLV-1/2 infection is primarily based on detecting specific IgG antibodies in serum using ELISA and chemiluminescent assays such as CLIA or ECLIA (26–28). Several commercially available kits built on recombinant Env and Gag HTLV proteins and/or synthetic peptide antigens alone or in combination with viral lysates were employed for global HTLV-1/2 antibody screening. Nonetheless, considering the significant homology between the two most common circulating HTLV types, several of those diagnostic assays fail to segregate infection by HTLV-1 and HTLV-2 in co-endemic areas (28, 29).

In this sense, only WB and qualitative and/or quantitative PCR are able to perform a type-specific diagnosis of HTLV-1 and HTLV-2 infections (29). Although WB is the most consolidated method for confirming and typing HTLV infection, it is not an automated method. Multiple sample testing in large scale represents a major concern for laboratory centers (30). Moreover, WB-indeterminate or non-typed results are commonly reported (31–33). Besides that, the high cost and the lack of universal standard operating procedure for molecular methods have restricted the wide use of qPCR for differential diagnosis of HTLV-1/2 infections, remaining applicable mostly in reference laboratories.

Considering these impairments in HTLV diagnostic methods, various countries that perform universal screening and differential diagnosis of HTLV-1/2 infections are raising the debate in and around the cost-effectiveness of such complex and stepwise diagnostic approaches. In general, the strategies proposed for differential diagnosis of HTLV-1 and HTLV-2 infections require the use of multiple assays using distinct serological and molecular platforms. Recently, Ji and collaborators performed a study that aimed at evaluating screening and confirmatory assays for the diagnosis of HTLV-1/2 infection (34). Several methods for screening of anti-HTLV-1/2 antibodies were compared (Avioq-ELISA, Murex-ELISA, Roche-ECLIA, and Fujirebio-CLIA) using individual plasma samples, and employing WB and LIA as gold standard serological tests followed by qPCR to confirm infection (34). The chemiluminescent assay demonstrated the highest performance; however, combination with the well-known ELISA serological test was needed to achieve the highest sensitivity. It is noteworthy that the LIA assay employed for confirming HTLV-1 and HTLV-2 infections yielded more indeterminate results as compared to the conventional WB assay (34). These findings emphasize the need of using multiple-step protocols to achieve the differential diagnosis of HTLV-1 and HTLV-2 infections. In this scenario, the search for a single-step approach for differential laboratorial diagnosis of HTLV-1/2 is a challenge for the scientific community. Previous studies using conventional indirect immunofluorescence assay (IFA) over glass slides on light microscopy has already been proposed for differential diagnosis of HTLV-1/2 infections (35, 36). These authors have shown that sensitivity of the IFA for HTLV-1/2 using glass slides by optical microscopy reached higher performance when the samples were tested simultaneously against both MT-2 and MoT antigens.

In the present study, we have developed an innovative flow cytometry assay, referred to as “FC-Duplex IgG1 (HTLV-1/2)” for universal and differential diagnosis of HTLV infections. The method herein proposed is a single-step optimized serological procedure for both universal and differential diagnosis of HTLV-1 and HTLV-2 infections. Our results demonstrated the high accuracy of the method in identifying HTLV-1 and HTLV cases using an automated high-throughput screening method.

To achieve the single competitive immunofluorescence platform by flow cytometry, a differential fluorescent labeling protocol was designed for HTLV-1 (MT-2) and HTLV-2 (MoT) cell lines as a specific antigenic solid support, using FITC and Alexa Fluor 647 dyes. Differential fluorescent labeling protocol with Alexa Fluor 647 or FITC free dyes can be achieved by using distinct concentrations since the fluorescence intensity emitted by any of these fluorescent dyes is proportional to the number of molecules bound to the target cells. Alexa Fluor 647 and FITC usually bind to proteins leading to stable staining. In the present protocol, we have used two different concentrations of the same fluorescent dye to label the MT-2 and MoT cell lines that could be further identified by the resultant fluorescent brightness, i.e., one cell line (MT-2) displayed low fluorescence intensity and the other (MoT) exhibited higher fluorescence intensity. The antigenicity of MT-2 and MoT cell lines was also evaluated and remained stable up to 12 months at −20°C. Differential staining of antigenic support with FITC or Alexa Fluor 647, on a single flow cytometric platform has already been reported by our group to accomplish the differential serological diagnosis of Chagas disease and leishmaniasis, based on species-specific anti-Trypanosomatidae IgG1 reactivity (37). In that work, Teixeira-Carvalho et al. (37) have also evaluated the stability of single and mixed Trypanosomatidae cell suspensions stained either with FITC or with Alexa Fluor 647, under various storage conditions. These authors reported that pre-stained Trypanosomatidae cell suspensions remained fluorescently stable for at least 1 year, with preserved differential brightness and antigenicity under distinct storage conditions. However, the authors observed that premixed labeled Trypanosomatidae cell suspensions exhibited considerable overlapping after 1 year of storage under different conditions. Therefore, based on the results of the present study, we recommend that pre-labeled MT-2 and MoT cell lines should be stored in separate batches and the MT-2 and MoT mix should be prepared immediately before use. Overall, our findings reinforce the idea that the “FC-Duplex IgG1 (HTLV-1/2)” reagents present staining and antigenic stability over storage time, fulfilling one relevant criterion for long-term storage of reagents, required for clinical assays.

Another relevant aspect of the “FC-Duplex IgG1 (HTLV-1/2)” resides on its design coupled with a high sensitivity development system (Biotin/Streptavidin/Phycoerythrin) that guarantee higher detection of IgG1 binding by flow cytometry, even using high serum dilutions.

Two parallel protocols of “FC-Duplex IgG1 (HTLV-1/2)” have been tested, referred to as “FIX” and “FIX & PERM”. Previous studies from our group, originally describing the innovative approach to detect anti-HTLV-1 IgG1 antibodies by flow cytometry, have tested the performance of a simplex protocol, using only the MT-2 cell line, applied to the diagnosis of HTLV-1 infection (20). The simplex system has been tested using both “FIX” and “FIX & PERM” protocols, demonstrating excellent performance for the diagnosis of HTLV-1 infection. However, a panoramic snapshot provided revealed that the “FIX & PERM” protocol presented higher sensitivity for detecting seropositivity. Our results demonstrated that overall, the performance of “FC-Duplex IgG1 (HTLV-1/2)” was higher using the “FIX” protocol for both universal and differential diagnosis of HTLV-1 and HTLV-2 infections. We assumed that while using a simplex non-competitive assay for detection of anti-HTLV-1 IgG1, the higher availability of antigenic epitopes provided by the fixation and permeabilization procedures using the “FIX & PERM” protocol enhances the seropositivity of MT-2 cells. Conversely, in the FC-Duplex IgG1 (HTLV-1/2) competitive platform, the mutual availability of both HTLV-1 and HTLV-2 antigenic epitopes simultaneously in MT-2 and MoT using the “FIX & PERM” protocol led to the waning IgG1 binding to both cell lines. Ultimately, the shared reactivity to common HTLV epitopes on MT-2 and MoT yielded lower reactivity as compared to the “FIX” protocol. The cross-reactivity of serum samples from HTLV-infected patients with MT-2 and MoT cells was expected. In fact, this is one of the limitations for using conventional slide immunofluorescence for differential HTLV-1/2 diagnosis since the conventional methods require the use of low serum dilutions and rely on the detection of total IgG, not IgG1, as used in the FC-Duplex IgG1 (HTLV-1/2). The flow cytometry utilized in the proposed method allows the use of higher serum dilutions, which makes it possible to find an optimal dilution to discriminate the reactivity of serum samples from HTLV-1 and HTLV-2 patients properly.

Ultimately, the use of synchronous and asynchronous pairwise analysis has improved the performance of “FC-Duplex IgG1 (HTLV-1/2)” for differential diagnosis of HTLV infection. These pairwise analyses, especially in the asynchronous pairs using a detuned assay, have contributed to obtain the selective reactivity to MT-2 and MoT cell lines at specific serum dilutions, leading to higher specificity.

Detuned serological approaches based on algorithms employing distinct serum dilutions have previously been proposed for serological diagnosis of infectious diseases (37–39). The concept of detuned assay was first described in 1998 by Janssen et al. (40). The detuned approach was originally termed “sensitive/less sensitive testing strategy” and designed for studies of HIV infection. The detuned assay procedure involves dual testing of the same sample with the same immunoassay. At first, the test is performed with a standard protocol, and secondly, samples are submitted to the same immunoassay, but using increased sample dilutions. Keeping this principle in mind, we have previously proposed a novel strategy for detuned assays applicable to serological diagnosis of Leishmaniasis and Chagas disease (37–39). One strategy was based on the use of stepwise or inverted detuned algorithms or applying distinct cutoff values at distinct serum dilutions. For the “FC-Duplex IgG1 (HTLV-1/2)” method, the detuned approach was based on the use of synchronous and asynchronous pairwise analysis to achieve the differential diagnosis of HTLV-1 and HTLV-2 infections. This strategy yielded outstanding reliability on the differential HTLV-1/-2 identification. Additionally, the assessment of specificity and sensitivity employing a panel of samples with low antibody levels that presented barely detectable titers with other standard techniques would provide further evidence about the performance of the “FC-Duplex IgG1 (HTLV-1/2)” method. Recently, Woo and colleagues have proposed a non-invasive method to detect antibodies to HTLV-1/2 using oral fluid, facilitating large-scale seroprevalence studies, enabling active surveillance of infection on a population level (41). In this sense, it might be interesting to test the potential of FC-Duplex IgG1 (HTLV-1/2) using antibodies from saliva.

Flow cytometry is a paramount technique in deep cell analysis, and it has been employed for many years in clinical practice for the diagnosis and monitoring of HIV and cancer patients as well as other autoimmune diseases. As flow cytometry has progressed, more applications and methods have been created for the benefit of diagnosis and clinical management of chronic diseases. From simple white blood cell counts to advanced flow-based techniques, flow cytometric methods can identify and unravel critical disease-related features in a fast-track manner, thereby allowing for early prevention and anticipation of patient needs (42). Flow cytometric equipment facilities are nowadays mandatory in several health institutes and hematological centers, which increases the applicability of flow-based methods such as the FC-Duplex IgG1(HTLV-1/2). An additional feature of flow-based methods is the ability of this technique to provide continuous numeric results such as percentages and mean and median fluorescence intensity, which is crucial for measuring quantitative analytes, antibody reactivity and cells in human samples. Considering that point-of-care rapid tests are yet to be available for HTLV-1 infection, this method could act as a surrogate screening test. However, a far from being a point-of-care diagnostic method, it is important to mention that FC-Duplex IgG1(HTLV-1/2) is designed as a method for confirmation of HTLV infection and viral typing after screening tests.

In conclusion, the present study has provided a proof of concept of the “FC-Duplex IgG1 (HTLV-1/2)” method using 135 well-characterized serum samples from patients with positive serology for HTLV-1/2 and non-infected controls. The proposed method presented an outstanding overall accuracy of 100% correct results for HTLV-1 and HTLV-2 differential diagnosis. In future experiments, we will validate the performance of “FC-Duplex IgG1 (HTLV-1/2)”, which is currently under investigation to compare the performance of intra- and inter-laboratorial analysis as well as the reproducibility using the proposed protocol and data generated by independent analysts.

## Data Availability Statement

The raw data supporting the conclusions of this article will be made available by the authors, without undue reservation.

## Ethics Statement

The studies involving human participants were reviewed and approved by the Research Ethics Committee of the René Rachou Institute - FIOCRUZ/MG (C.A.A.E. N° 15047313.8.0000.5091). The patients/participants provided their written informed consent to participate in this study.

## Author Contributions

LP, JC-d-R, VP-M, BT, JM, LA, AC-P, ES, KB, AT-C, and OM-F designed the study, performed the experiments, and analyzed the data. LP, JC-d-R, VP-M, KB, AT-C, and OM-F wrote and reviewed the full manuscript. All authors contributed to the article and approved the submitted version.

## Funding

The study has been supported by Fundação de Amparo à Pesquisa do Estado de Minas Gerais (PPSUS/FAPEMIG Grant # APQ-00821-20). MS, AT-C, JC-d-R, and OM-F received PQ Fellowship from Conselho Nacional de Desenvolvimento Científico e Tecnológico CNPq. AT-C and OM-F are also grateful to the FAPEAM (PVN-II, PRÓ-ESTADO Program #005/2019) for the research fellowship program. JM received PIBIC fellowship from CNPq.

## Conflict of Interest

The authors declare that the research was conducted in the absence of any commercial or financial relationships that could be construed as a potential conflict of interest.

## Publisher’s Note

All claims expressed in this article are solely those of the authors and do not necessarily represent those of their affiliated organizations, or those of the publisher, the editors and the reviewers. Any product that may be evaluated in this article, or claim that may be made by its manufacturer, is not guaranteed or endorsed by the publisher.

## Supplementary Material

Supplementary Figure 1 Applicability of “FC-Duplex IgG1 (HTLV-1/2)” assay for prognosis of HTLV-1/2 infection. **(A)** Panoramic overview of “FIX” and “FIX & PERM” protocols according to the overall anti-MT-2 and anti-MoT IgG1 mean reactivities along the titration curves (1:32 to 1:4,096) used to identify the target cell line and the serum dilution with higher segregation score (Δ = delta reactivity) between HAC vs HAM groups (dashed rectangle). **(B)** Overall performance of “FC-Duplex IgG1 (HTLV-1/2)” assay for prognosis of HTLV-1 infection assessed by scatter plot distribution of individual values of IgG1 reactivity, TG-ROC parameters and ROC curve analysis using the selected cell line and pair of attributes “serum dilution/cut-off” (MT-2 1:256/PPFC=60%) from the “FIX” protocol. Scatter plot distribution of IgG1 reactivity profile of samples from patients with putative HAM status (pHAM) as compared to HAC and HAM.
