# Supplementary figures and images for: A New Flow Cytometry-Based Single Platform for Universal and Differential Serodiagnosis of HTLV-1/2 Infection
Source: Front Immunol. 2022 Apr 14;13:795815. doi: 10.3389/fimmu.2022.795815 (PMC9047958; doi:10.3389/fimmu.2022.795815)

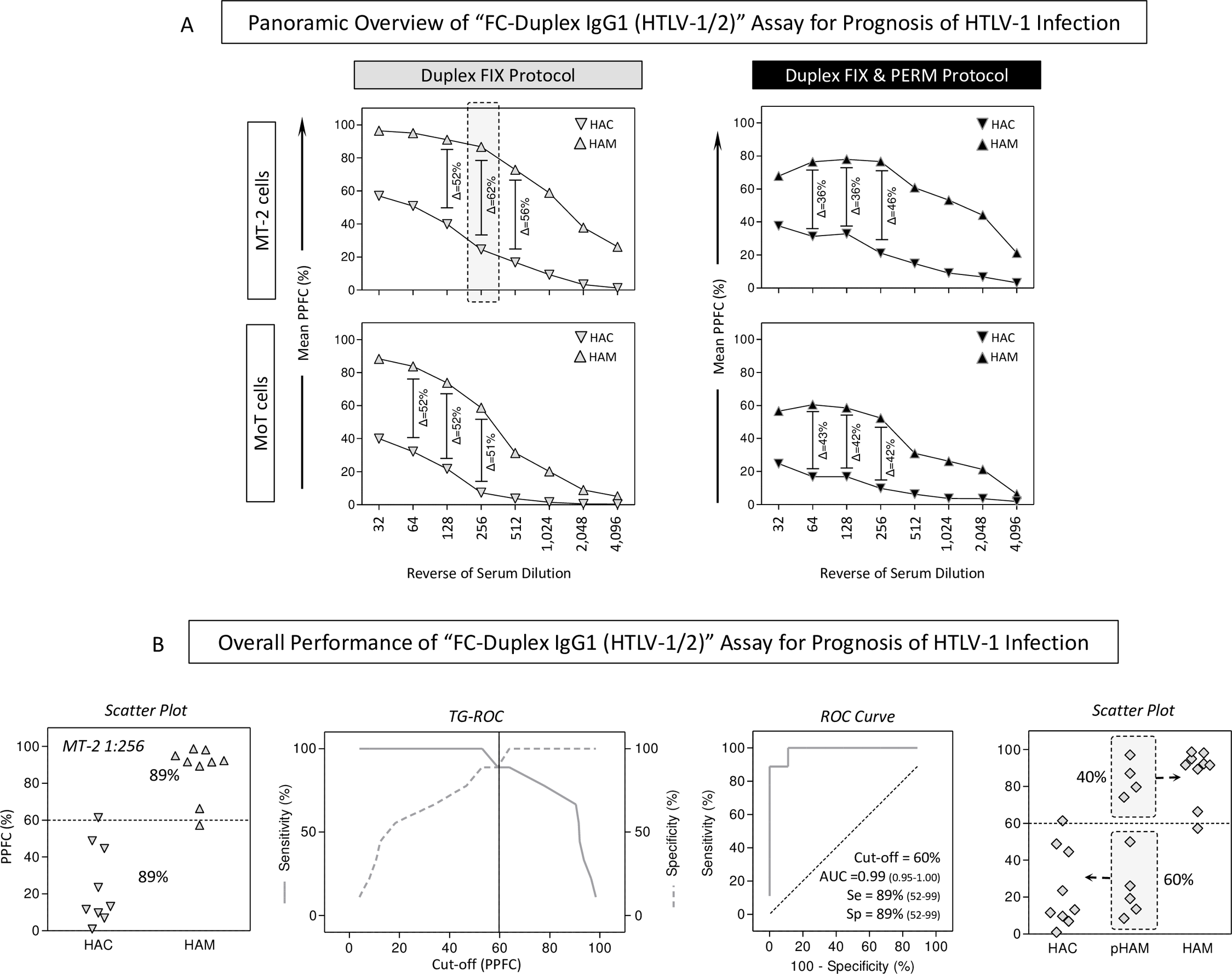

Supplement: Supplementary Figure 1 — Applicability of “FC-Duplex IgG1 (HTLV-1/2)” assay for prognosis of HTLV-1/2 infection. (A) Panoramic overview of “FIX” and “FIX & PERM” protocols according to the overall anti-MT-2 and anti-MoT IgG1 mean reactivities along the titration curves (1:32 to 1:4,096) used to identify the target cell line and the serum dilution with higher segregation score (Δ = delta reactivity) between HAC vs HAM groups (dashed rectangle). (B) Overall performance of “FC-Duplex IgG1 (HTLV-1/2)” assay for prognosis of HTLV-1 infection assessed by scatter plot distribution of individual values of IgG1 reactivity, TG-ROC parameters and ROC curve analysis using the selected cell line and pair of attributes “serum dilution/cut-off” (MT-2 1:256/PPFC=60%) from the “FIX” protocol. Scatter plot distribution of IgG1 reactivity profile of samples from patients with putative HAM status (pHAM) as compared to HAC and HAM. [file Image_1.tif]
